# Psychodermatology special edition

**DOI:** 10.1002/ski2.102

**Published:** 2022-02-14

**Authors:** G. W. M. Millington

**Affiliations:** ^1^ Dermatology Department Norfolk and Norwich University Hospital Norwich UK

1

Skin Health and Disease (SHD) was launched in September 2020. It was the first new journal from the British Association of Dermatologists in over 100 years.^1^, ^2^, ^3^ Psychodermatology is the crossover discipline between Dermatology and Clinical Psychology and/or Psychiatry. It includes equally Psychiatric diseases that present with skin symptoms and signs (such as delusional infestation) or more commonly, the psychiatric or psychological problems associated with dermatological illness, such as depression associated with psoriasis.^4^ Since the journal was launched, I noticed that we had had quite a few manuscripts submitted that would definitely fit under the category of predominantly Psychodermatology or Dermatology manuscripts with a psychological overlay.^4^, ^5^, ^6^, ^7^, ^8^, ^9^, ^10^, ^11^ The editorial team of SHD will launch a themed issue later in the year on the subject of Psychodermatology. I am extremely grateful to Prof Tony Bewley and Dr Susannah Baron (London) and Prof John Koo and Dr Marwa Hakimi (San Francisco) for agreeing to be guest editors. If you want to discuss a Psychodermatology submission with me, or any of the guest editors, please email shd@bad.org.uk.

## CONFLICT OF INTEREST

G. W. M. Millington is the Editor in Chief of Skin Health and Disease.

## AUTHOR CONTRIBUTIONS

**G. W. M. Millington:** Writing – original draft.

## Data Availability

There are no original data. This is an editorial.
